# Correction: Köksal et al. Interplay Between HIV and Human Pegivirus (HPgV) Load in Co-Infected Patients: Insights from Prevalence and Genotype Analysis. *Viruses* 2024, *16*, 5

**DOI:** 10.3390/v16121883

**Published:** 2024-12-05

**Authors:** Muammer Osman Köksal, Martin Pirkl, Kutay Sarsar, Mehmet Ilktaç, Gibran Horemheb-Rubio, Murat Yaman, Sevim Meşe, Haluk Eraksoy, Baki Akgül, Ali Ağaçfidan

**Affiliations:** 1 Department of Medical Microbiology, Istanbul Faculty of Medicine, Istanbul University, Istanbul 34093, Türkiye; kutay.sarsar@istanbul.edu.tr (K.S.); murat.yaman@istanbul.edu.tr (M.Y.); smese@istanbul.edu.tr (S.M.); ali.agacfidan@istanbul.edu.tr (A.A.); 2 Institute of Virology, Faculty of Medicine and University Hospital of Cologne, University of Cologne, 50935 Cologne, Germany; martin.pirkl@uk-koeln.de (M.P.); gibran.rubio@pei.de (G.H.-R.); baki.akguel@uk-koeln.de (B.A.); 3 Department of Pharmaceutical Microbiology, Faculty of Pharmacy, Eastern Mediterranean University, Famagusta 99450, Cyprus; mehmet.ilktac@emu.edu.tr; 4 Department of Infectious Disease and Microbiology, Istanbul Faculty of Medicine, Istanbul University, Istanbul 34093, Türkiye; eraksoyh@istanbul.edu.tr

## Text Correction

In the original publication [1], the authors were unaware that a thesis had already been published (Ebru Yücebağ. Investigation of frequency and genotype diversity of human pegiviruses (HPgV) in people infected with human immunodeficiency virus (HIV), Yüksek Lisans tezi, İstanbul Üniversitesi-Cerrahpaşa, Türkçe, 2020), leading to the removal of the following inappropriate statements: the last sentence in the Introduction Section, “To date, no study has been conducted on HPgV in HIV-infected patients in Türkiye”; the first sentence in the first paragraph of the Discussion Section, “To the best of our knowledge, this present study represents the first comprehensive investigation into the prevalence of HPgV in HIV-infected patients in Türkiye”; and the first sentence in the first paragraph of the Conclusions Section, “In conclusion, this study represents the first comprehensive investigation of the prevalence of HPgV and its impact on HIV-infected individuals in Türkiye”.

The authors state that the scientific conclusions are unaffected. This correction was approved by the Academic Editor. The original publication has also been updated.
