# Supplementary material for: Interplay Between HIV and Human Pegivirus (HPgV) Load in Co-Infected Patients: Insights from Prevalence and Genotype Analysis
Source: Viruses. 2023 Dec 19;16(1):5. doi: 10.3390/v16010005 (PMC10820514; doi:10.3390/v16010005)
Supplement: Supplementary file 1 [file viruses-16-00005-s001.zip › Figure S1.pdf]

viruses

MDPI

Article

Supplement: Interplay between HIV and Human Pegivirus (HPgV) Load in Co-Infected Patients: Insights from Prevalence and Genotype Analysis

Muammer Osman Köksal [1,*], Martin Pirkl [2], Kutay Sarsar [1], Mehmet Ilktaç [3], Gibran Horemheb-Rubio [2], Murat Yaman [1], Sevim Meşe [1], Haluk Eraksoy [4], Baki Akgül [2,†] and Ali Ağaçfidan [1,†]

[1] Department of Medical Microbiology, Istanbul Faculty of Medicine, Istanbul University, Istanbul 34093, Türkiye; kutay.sarsar@istanbul.edu.tr (K.S.); murat.yaman@istanbul.edu.tr (M.Y.); smese@istanbul.edu.tr (S.M.); ali.agacfidan@istanbul.edu.tr (A.A.)

[2] Institute of Virology, Faculty of Medicine and University Hospital of Cologne, University of Cologne, 50935 Cologne, Germany; martin.pirkl@uk-koeln.de (M.P.); gibran.rubio@pei.de (G.H.-R.); baki.akguel@uk-koeln.de (B.A.)

[3] Department of Pharmaceutical Microbiology, Faculty of Pharmacy, Eastern Mediterranean University, Famagusta 99450, Cyprus; mehmet.ilktac@emu.edu.tr

[4] Department of Infectious Disease and Microbiology, Istanbul Faculty of Medicine, Istanbul University, Istanbul 34093, Türkiye; eraksoyh@istanbul.edu.tr

* Correspondence: muammerosmankoksal@istanbul.edu.tr

† These authors contributed equally to this work.

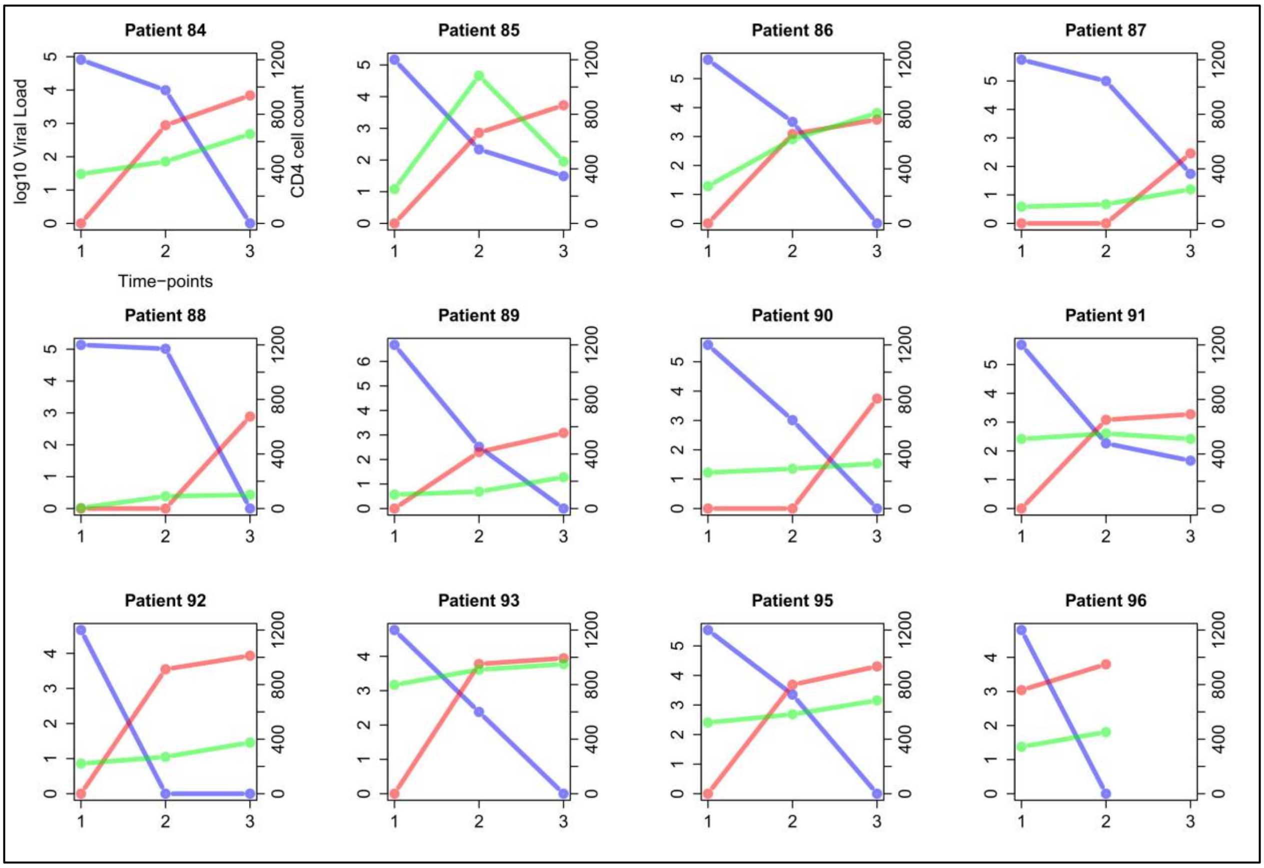

Figure S1. Per patient view of time points corresponding to Figure 3 (blue: HIV viral load; red: HPgV viral load; green: CD4 cell count).
